# Proactive and Ongoing Analysis and Management of Ethical Concerns in the Development, Evaluation, and Implementation of Smart Homes for Older Adults With Frailty

**DOI:** 10.2196/41322

**Published:** 2023-03-09

**Authors:** Rosalie H Wang, Thomas Tannou, Nathalie Bier, Mélanie Couture, Régis Aubry

**Affiliations:** 1 Department of Occupational Science and Occupational Therapy Temerty Faculty of Medicine University of Toronto Toronto, ON Canada; 2 Rehabilitation Sciences Institute Temerty Faculty of Medicine University of Toronto Toronto, ON Canada; 3 Centre de recherche de l’Institut universitaire de gériatrie de Montréal Centre intégré universitaire de santé et de services sociaux (CIUSSS) - Centre-Sud-de-l’île-de-Montréal Montréal, QC Canada; 4 Centre d’Investigation Clinique de l’Institut National de la Santé et de la Recherche Médicale (INSERM CIC) 1431 Centre Hospitalier Universitaire de Besançon Besançon France; 5 Programme d’ergothérapie, École de réadaptation Faculté de médecine Université de Montréal Montréal, QC Canada; 6 École de Travail social Université de Sherbrooke Sherbrooke, QC Canada

**Keywords:** ethics, older adults, frailty, smart home, assistive technology, aging in place, ethical concerns, implementation, bioethics, technology ethics, autonomy, privacy, security, informed consent, support ecosystem

## Abstract

Successful adoption and sustained use of smart home technology can support the aging in place of older adults with frailty. However, the expansion of this technology has been limited, particularly by a lack of ethical considerations surrounding its application. This can ultimately prevent older adults and members of their support ecosystems from benefiting from the technology. This paper has 2 aims in the effort to facilitate adoption and sustained use: to assert that proactive and ongoing analysis and management of ethical concerns are crucial to the successful development, evaluation, and implementation of smart homes for older adults with frailty and to present recommendations to create a framework, resources, and tools to manage ethical concerns with the collaboration of older adults; members of their support ecosystems; and the research, technical development, clinical, and industry communities. To support our assertion, we reviewed intersecting concepts from bioethics, specifically principlism and ethics of care, and from technology ethics that are salient to smart homes in the management of frailty in older adults. We focused on 6 conceptual domains that can lead to ethical tensions and of which proper analysis is essential: privacy and security, individual and relational autonomy, informed consent and supported decision-making, social inclusion and isolation, stigma and discrimination, and equity of access. To facilitate the proactive and ongoing analysis and management of ethical concerns, we recommended collaboration to develop a framework with 4 proposed elements: a set of conceptual domains as discussed in this paper, along with a tool consisting of reflective questions to guide ethical deliberation throughout the project phases; resources comprising strategies and guidance for the planning and reporting of ethical analysis throughout the project phases; training resources to support leadership, literacy, and competency in project teams for the analysis and management of ethical concerns; and training resources for older adults with frailty, their support ecosystems, and the public to support their awareness and participation in teams and ethical analysis processes. Older adults with frailty require nuanced consideration when incorporating technology into their care because of their complex health and social status and vulnerability. Smart homes may have a greater likelihood of accommodating users and their contexts with committed and comprehensive analysis, anticipation, and management of ethical concerns that reflect the unique circumstances of these users. Smart home technology may then achieve its desired individual, societal, and economic outcomes and serve as a solution to support health; well-being; and responsible, high-quality care.

## Introduction

Population aging, along with chronic disease and disability among older adults, are increasing challenges for supporting a high quality of life and sustaining services in health and social care systems. The COVID-19 pandemic has highlighted the many limitations of multiple health systems worldwide whose delivery models struggled to keep up with service demands. With widespread calls for physical distancing to limit COVID-19 transmission, the existing social isolation of older adults [1,2] has been exacerbated. However, the pandemic has enabled an unprecedented surge in the use of new technologies in all parts of society, particularly in health systems. Nevertheless, some solutions are slow to be adopted, particularly those based on smart home technology. Often situated in a private home, a smart environment “adopts ICT [information and communication technology] to collect and share information, analyze and monitor residents’ behavioral patterns, and improve residents’ quality of life” [3]. Smart homes entail a combination of products and services that make up a smart environment (also referred to as active and assisted living systems). In addition to being, for the most part, at a low level of maturity and with little strong evidence of effectiveness [4-7], smart home technology may be slowly adopted because of unmitigated ethical concerns [4].

This viewpoint paper has 2 aims in the effort to facilitate the adoption and sustained use of smart home technology: (1) to assert that proactive and ongoing analysis and management of ethical concerns are crucial to the successful development, evaluation, and implementation of smart homes for older adults with frailty and (2) to present recommendations to create a framework, resources, and tools to manage ethical concerns with the collaboration of older adults; members of their support ecosystems; and the research, technical development, clinical, and industry communities.

The paper is structured as follows. We begin by explaining the nature of frailty and the importance of addressing it. We discuss smart home technology and its potential to support older adults with frailty. Our work summarizes key anthropological concepts that are relevant to smart homes when managing frailty in older adults and how these concepts can lead to ethical concerns. Thus, we discuss the rationale for proactive and ongoing analysis and management of these ethical concerns from development to sustained use. Finally, we present recommendations and opportunities for collective action to create and implement a framework, resources, and tools.

## Background

### Older Adults With Frailty

Approximately 32 million Europeans [8] and 1.6 million Canadians [9] are estimated to experience frailty. Frailty is an umbrella term that encapsulates a constellation of conditions with varying severity and consequences for individual older adults. It presents with poor health and function and heightened vulnerability to incidental adverse health events and deteriorating quality of life [10]. The integrated model consensually developed by Gobbens et al [11] highlights 3 major components of frailty—physical, psychological, and social—that dynamically interact to create this situation of vulnerability. For example, a diagnosis of frailty may be assigned if physical conditions (eg, malnutrition or mobility restrictions because of arthritis) that may affect or be affected by psychological capacity (eg, cognitive decline caused by Alzheimer disease or low mood because of depression) co-occur with an unsupportive social situation (eg, poor social relations or isolation). In such a scenario, an adverse event such as a fall is more likely to occur and may cause an older adult to reduce social activities and self-isolate at home. A vicious cycle may thereby be created and further increase the risk of falls and physical, psychological, and social vulnerability. Without intervention, interactions between these frailty components may lead to the following: (1) disability or difficulties performing self-care and household management activities, creating dependency; (2) falls and mobility reduction; (3) hospitalizations; (4) changes in living situations (eg, moving to a long-term care home); and (5) death. However, frailty, depending on the modifiability and interactions of the diseases and conditions, may be preventable and reversible with appropriate interventions [12].

Research suggests that frailty may be viewed as progressive alterations in an older adult’s intrinsic capacities (ie, motor skills, cognition, and sensory functions), suggesting reversibility through strengthening of these altered capacities with health and social interventions (eg, physical and cognitive rehabilitation and participation in valued social and community activities) [13]. As such, it is imperative to identify frailty (or the risk of frailty) and implement early intervention. However, with persistent impairments, support strategies may be based on compensation for the loss of capacity and function to reduce dependency [14]. Social support and technology may be crucial environmental elements in early detection and intervention [5,15]. Digital solutions such as smart home technology may play key roles in mitigating some of the causes and consequences of social isolation and frailty [16] while respecting older adults’ right to self-determination.

### Smart Homes

Smart homes may be interventions to address pressing concerns about supporting the high quality of life of older adults, relieve stressors on informal supports, and mitigate the challenges of health and social system sustainability with population aging. Users of smart homes may include older adults and members of their support ecosystems (ie, family or friends in caregiving roles, physicians, therapists, social workers, nurses, and home support workers) [17]. (Additional users necessarily include the research, technical development, and industry communities as they are responsible for data maintenance and support for use.) The combination of smart home technology and human support constitutes a powerful environmental support strategy. Indeed, these homes may be viewed as an embodiment of the collective human intelligence. This collective intelligence may support shared goals and respect the older adult and members of their support ecosystem. This may be done by supporting decision-making processes and affording efficient, personalized, and adaptive management of frailty in older adults living at home.

Smart home technology may assist with daily activities in various ways. Broad functions may include home environment or appliance automation (eg, thermostat control and stove shutoff), health or activity tracking for self-management purposes, monitoring and alerting for safety or emergency situations, and supporting social connectedness [17,18]. Sensors embedded in a home may collect longitudinal data to monitor a resident’s health status, behaviors, and activities. Important frailty-related information regarding cognition, mobility, and daily activity performance status may be monitored and inferred through activity patterns and indicators [5,15]. Data integration and analysis using artificial intelligence (AI) approaches such as machine learning may allow for the creation of tools that predict or detect events. The detection of adverse events such as falls or concerning trends in health, such as a routine disruption that may suggest the occurrence of a delirium, may expedite access to intervention from caregivers and health care providers. Reminders or stepwise assistance to perform daily activities may be delivered based on the system’s input, observed, or learned knowledge of residents’ behaviors, activity patterns, and preferences. Residents may interact with a smart home through various interfaces involving voice, touch, motion, or gestures [19]. Although many specialized smart home products and services are in development for older adults with health needs [4], there is also a proliferation of technology for general consumers that offers desirable functions for older adults to enhance environmental comfort (eg, automated control of lights or thermostat), safety (eg, home security systems and automated door locks), and daily activity performance (eg, kitchen appliances).

Despite innumerable smart home projects at various phases of development and implementation worldwide, the vast majority are not realized into commercial products and services, and mainstream technology on the market has not been widely adopted by older adults [18,20]. Ethical challenges have been reported to influence the successful adoption and sustained use of smart homes [21]. There is also increased focus on systematically considering ethics as part of the Health Technology Assessment that supports policy recommendations for available technology [19,22,23]. Nevertheless, little attention has been paid to ethical evaluation during the design of intelligent assistive technology such as smart homes [24,25]. Few researchers have highlighted the imperative for comprehensive inclusion of ethical considerations across processes for development, evaluation, implementation, and the sustained use of smart home technology. As such, we assert that proactive and ongoing analysis and management of ethical concerns are crucial to the successful development, evaluation, and implementation of smart homes for older adults with frailty.

## Concepts and Ethical Challenges

### Overview

Ethical challenges and the need for deliberation arise when anthropological concepts such as values conflict with other values that are considered equally important. Applied ethics is the field that examines real-world applications of this deliberation and action. We bring together concepts from often-siloed fields of bioethics, such as principlism and ethics of care, and technology ethics to highlight ethical challenges to be deliberated with respect to smart home technology.

### Principlism

Principlism by Beauchamp and Childress [26] refers to a dominant framework in bioethics that outlines 4 key principles—respect for autonomy, beneficence, nonmaleficence, and justice. Although the latest edition of Beauchamp and Childress [26] includes discussions of moral virtues and features of the professional-patient relationship, such as veracity, privacy, confidentiality, and fidelity, the 4 principles remain pillars of bioethics. Autonomy is the fundamental right to make decisions about oneself and to do what one chooses within one’s own life. A distinction can be made between being autonomous and independent. Being autonomous means being able to express and act in terms of one’s own free will, which may include choosing to receive assistance from others. Being independent means not requiring assistance from others. Beneficence relates to the responsibility to act in ways that benefit others overall, which may include preventing, mitigating, and removing harm to others and promoting benefits. Nonmaleficence refers to the responsibility to abstain from or avoid actions that cause or may cause harm to others. Justice is the principle that is concerned with fairness (treating everyone with the same concern and respect) and equity (seeing that benefits and harms are distributed across people as equals) [27].

### Ethics of Care

Although ethics of care, or care ethics, may be positioned as an alternative to the rationalist principle-oriented perspective of principlism [28], it is valuable to consider both approaches as they are complementary when examining the management of frailty in older adults. Maio [28] concentrates on the core ideas of care ethics without invoking the historical gendered view of care and ethics. Fundamentally, the ethics of care deals with the asymmetric nature of care relationships and the potential dependency and vulnerability that can result. Furthermore, assuming that care relationships are, to varying degrees, asymmetrical does not deny the equality of people in care-associated relationships and the sovereignty of individuals to determine their life and care goals. On the contrary, it requires all participants to appraise this vulnerability to offer adequate support. In this way, according to Maio [28], ethics of care must be situation-oriented; responsive; and informed by knowledge from experiences, relationships, and the situation at hand.

In details, according to the revised version of the 4-phase model of care by Tronto [29], ethics of care leads to acceptance of fundamental dependency on and need of mutual support (Conradi, 2001, as cited in Maio [28]). Attentiveness is the first step to be able to care about someone else. Keeping in mind the concern for the other leads to an inclination to respond to their needs. This response to another’s needs is grounded in a sense of responsibility and translates into direct action or chains of action depending on the competencies of caregivers and singularities of each situation and relationship. Individual competency and the ability to initiate the required chain of action to manage the situation are key. The ability to provide a solution or initiate a chain of solidarity to support an identified need is the basis of ethics of care.

### Technology Ethics

There is currently no dominant or unified approach to technology ethics. Ethical issues, implications, and decision-making practices arising from the design, use, and spread of different technologies are much-discussed topics in different fields and referred to using various terms (eg, technoethics, ethics of technology, computer ethics, AI ethics, and machine ethics). Consistent across these fields is the need to examine ethical, social, and legal implications, and there is often an orientation toward supporting citizens’ rights [30]. Many concerns are based on the rapid evolution of digital technology and AI, which raise new questions, possibilities, and challenges that require careful deliberation. Technological developments may have a range of intended and unintended uses and outcomes or may amplify existing societal problems. Indeed, care ethics, with its emphasis on personal relationships, may rightly or wrongly subordinate care delivered through technical means in relation to human care [30]. These developments require us to continuously reflect on and modify our individual and collective values and practices.

Concepts in technology ethics are numerous and often include those of bioethics. Friedman and Kahn [31] in the field of human-computer interaction outline 12 human values they consider to be ethically important in computer ethics. The 12 values are human welfare, ownership and property, privacy, freedom from bias, universal usability, trust, autonomy, informed consent, accountability, identity, calmness, and environmental sustainability. In the Institute of Electrical and Electronics Engineers Global Initiative on Ethics of Autonomous and Intelligent Systems, 5 general ethical principles were identified as critical in the design, development, and implementation of technology: human rights, well-being, accountability, transparency, and awareness of misuse [32].

## Ethical Challenges Related to Smart Homes for Older Adults With Frailty

### Overview

Bioethics and technology ethics concepts are salient but intersecting. Examining these concepts in relation to smart homes for use by older adults and in the management of frailty can lead to ethical challenges. We describe in depth 6 key conceptual domains that need to be analyzed for smart homes but that can lead to ethical tensions.

### Privacy and Security

Privacy and security concerns are multidimensional and often interrelated. There are 2 dimensions of privacy that warrant examination: privacy of personal information (ie, identity) and physical privacy (ie, related to one’s body and the activities and routines being carried out in different spaces) [24]. Security may refer to information security or the safeguarding of data from unauthorized access. In addition, security may pertain to the experience of safety or trust in someone or something in a situation.

Information privacy and security are linked as maintaining privacy necessitates the security of information. Ethical concerns relate to the vast amount of sensitive information (eg, personal, medical, physiological, behavioral, and locations) that may be collected from users, how and for what purposes the information may be used, and who has and should have access to the information (eg, family, friends, health care providers, insurers, and manufacturers). Furthermore, the loss of private and confidential information to crime or unauthorized or wrongful access and use may lead to safety concerns and loss of the feeling of security [19]. Of particular importance to older adults with cognitive impairments or who are dependent on others are the experiences of uncertainty regarding what information is being collected, who has access (both intentional and unintentional in the case of an information breach), whether access may be controlled by users or others, and how reliable and trustworthy are those who have access. In such cases, harm may be inflicted without the older adult’s knowledge or control.

Privacy concerns are commonly voiced by older adults when considering smart homes and their adoption [18,20,33]. Facilitating personal safety and feelings of security is important for living at home autonomously and may be a strong motivator for adoption. There may need to be a trade-off between gaining security (and, thereby, freedom) and the loss of privacy if activities or routines in the home are monitored [19]. When older adults and those in their support ecosystems are trying to enhance safety and security with remote monitoring (eg, installation of cameras or other sensors in the bathroom to alert for assistance after a fall), a balance needs to be created when setting up systems to see that older adults’ privacy rights and personal wishes are respected. Older adults with cognitive impairments may need more monitoring as they may be at greater risk of harm [19] but less able to express their wishes.

Issues of privacy loss may be mediated by perceptions of the smart home technology’s usefulness. If the functions or services provided by the technology are perceived to be beneficial, older adults may elect to disregard some privacy concerns [24,33]. Some studies have reported that people with better health prioritize privacy protection more than those with poorer health and that those with poorer health prioritize the potential benefits of technology use [18]. However, there may be a tendency for older adults to favor conservative options when presented with novel, uncertain, or even risky situations [34]. That is, in making decisions, they may elect to implement strategies aimed at avoiding losses rather than optimizing gains. This prospect may need to be accounted for in decision-making regarding the adoption and use of smart home technology. There may also be scenarios in which older adults with cognitive impairment do not perceive a risk and feel safe despite the heightened risk related to frailty. In such cases, they may reject technological assistance based on its perceived nonusefulness. These trade-offs among the need to maintain privacy standards, minimize losses, and accept benefits necessitate heightened awareness to ensure that older adults who may be vulnerable are not exploited and able to make the best decision for their situation.

### Individual and Relational Autonomy

An important dimension of autonomy involves older adults’ relationships with others in their support ecosystems, as underscored in care ethics. Individual autonomy is the right of individual older adults to make choices about their lives and act freely without external influences [26]. However, in care contexts, relational aspects are critical, whether they are with other people or environmental elements such as smart homes [35,36]. In relational autonomy, dynamic interactions between individuals and others around them cocreate an individual’s identity, interests, and needs. The reality when considering respect for autonomy is that decisions of older adults regarding how they are supported (eg, by other people, technology, or a combination of these), what support they accept and when, what benefits are desirable, or what risks or harms are acceptable are affected by and will affect those around them. For instance, in cohabitation situations where remote home monitoring is considered, decisions affect others directly, so collective privacy needs to be discussed [30].

Some research has identified that caregivers perceive the benefits of technology use more than older adults who feel they may do without technological support [18]. Family members have reported feeling trust in the technology and that it would help their older relatives carry out more activities on their own, whereas older adults felt that smart homes may help in emergency situations but reported feeling more secure with another person present [17]. Overemphasis on either individual or relational perspectives has been critiqued as one may risk neglecting collective decision-making to benefit individuals and the other may neglect individual older adults’ needs over those of others. The relational influences of ethical import on the adoption and use of smart homes for older adults with frailty and enhanced vulnerability may include positive and negative social pressure, persuasion, or even coercion from family, friends, or other social forces [18].

Beyond autonomy in care relationships with other people, ethical concerns have been raised regarding relationships with technology in the management of health and social needs. Concerns have been identified over the loss of individual autonomy with technology use in circumstances where it is perceived to control what older adults do or provide too much assistance [19]. Fears may be experienced by older adults and those in their support ecosystems that they may become overreliant on technology or automation [19].

Respecting the autonomy of older adults and their support ecosystems individually and within relationships may necessitate the adoption of the tenets of care ethics and the application of a support ecosystem–centered approach. Information about and experience with using smart home systems needs to be personalized to support their collective goals and informed decision-making about use. Notably, older adults with cognitive impairments may require additional considerations for making their needs known and support in shared decision-making [37]. The original theory of discourse ethics by Habermas [38], which emphasized the imperative for intersubjectivity in arriving at moral standards [39], may be pertinent to decision-making in relational autonomy. The theory with key concepts adapted by Frantik [40] to be inclusive of people with dementia may be particularly useful as it affirms their rights and empowers them through practical strategies to participate equally in negotiations that result in decisions affecting their lives.

### Informed Consent and Supported Decision-making

Informed consent may only occur when people have knowledge and understanding of technology, their intended purposes and uses, and the potential benefits and harms the technology may create for them and their situations. A lack of awareness, familiarity, knowledge, and skills associated with smart home technology undermines consent capacity. This may have implications for adoption and use, the selection of options or features to maximize benefits and minimize risks during use, and consent to terms of service or data use [20,25,33]. These gaps may lead users to distrust technology or reject its use as they experience a loss of autonomy. Users may not even be aware of or comprehend the fact that these are ethical concerns [24].

Requirements for informed consent become more complex and unfeasible with the addition of AI to smart home technology because of limitations in the transparency and traceability of algorithmic decisions [25,30,41]. Transparency issues relate to how well users may understand how decisions are being made. With AI, large data sets are processed continuously and used to autonomously learn about users and make decisions to eventually carry out a desired action. Decision-making opportunities are not presented to users, so they do not have explicit choices, and it is unrealistic for users to make all these decisions. Algorithms tend to be opaque such that the decisions of the system cannot be explained [30]. Furthermore, algorithms evolve over time, whereby the functions and abilities of algorithms may no longer be consistent with those that users granted their consent for [25]. Although users are asked to consent to sharing a lot of their data, it may be challenging for users to assess benefits and harms. Indeed, developers who use the data and algorithms cannot adequately assess the benefits and harms as the exact ways in which decisions or actions are determined from the algorithms may be unknowable. Traceability refers to whether the cause of harm may be identifiable in situations where multiple actors are involved in the creation or implementation of an algorithm and who may be accountable and liable for harms [41].

Informed consent by older adults also depends on the availability of high-quality information. The proliferation of misinformation creates challenges in disentangling information and finding information from trustworthy and reliable sources. Unscrupulous or unknowledgeable individuals may also sell products or services targeted at older adults that overemphasize benefits and minimize potential harms. Considering older adults’ tendency to select options that minimize losses in novel circumstances, how or by whom information is presented may be more important than what is presented. Older adults, especially those with cognitive impairments or who are socially isolated, may be particularly vulnerable to mistreatment related to the trustworthiness of information.

Without accessible information or appropriate learning opportunities that are oriented to enhance understanding by older adults who may be unfamiliar with technology or have cognitive limitations, informed consent and decision-making may not be possible. The information offered to older adults may be overly simplistic (ie, merely asking for agreement to use a service) or complicated (ie, detailing specifics of terms of service) [24]. Even in the absence of disease, cognitive aging is associated with a decline in information-processing speed. As such, the ability to make decisions is preserved only when enough time and explanations are provided in an environment where distractions are minimized [34]. For some older adults with cognitive impairments, the availability of ongoing decision-making support may be essential across the spectrum of cognitive abilities. For example, trained and trusted family members, health or social care professionals, or substitute decision makers may be essential to explain information in understandable ways or grade decision-making to match the abilities of the older adult. Determining advanced directives for older adults with cognitive impairments may be a possible solution, although assent will continue to be required by assessing older adults’ verbal or nonverbal signs of agreement or disagreement in specific situations [25].

### Social Inclusion and Isolation

Commonly discussed functions of smart homes are to enhance social connectedness, support, and inclusion; reduce isolation through remote communication with caregivers or health care providers; and offer ready access to assistance in emergency situations. Given the anticipated shortage of health care providers and working-age caregivers and changes in family living arrangements, with younger generations living further away from senior family members, care from a distance through technology is increasingly the reality [42,43]. However, older adults have voiced concerns over the potential loss of social contact and human touch when care technologies such as smart homes are suggested [18-20,33]. With the capabilities of AI, there may be the added threat of replacing care providers and further compromising relationships [44].

Considering care ethics, relationships and experiences of empathy and responsiveness to needs are critical. As such, technology serves as an augmentative tool and one of several elements of personalized care. Overreliance on technology-based care, whereby technology is applied as a substitute for in-person interactions, may have detrimental effects in situations involving older adults with frailty. Nevertheless, a possibility may be that some older adults wish to include technology in their health management to limit social contact, preserve private time, or protect privacy [19]. Considering the social needs of older adults and members of their support ecosystems relates back to respecting autonomy in the management of frailty. Beneficence and maleficence also need examination to balance the overall benefits and harms of the use of smart homes as components of health and social care for those involved.

### Stigma and Discrimination

Stigma and discrimination are concerns that may vary across sociocultural contexts and result in considerable harm. These concerns are pertinent to older adults’ adoption and use of smart home technology and are important in the development and implementation of potentially beneficial technology. Stigma may be defined as “a set of negative and often unfair beliefs that a society or group of people have about something” [45]. Stigma is associated with being an older adult (ie, being *elderly* and senile) or having a disability and the perception of being a burden. Older adults have expressed concerns over the stigma associated with the technology used to support health and social care [18,33]. The use of such technology may be perceived to reflect diminished health and increased frailty and disability—characteristics with which older adults may not personally identify. The obtrusiveness of technology (eg, whether installed components or functions are clearly visible or audible to others and call attention to personal problems) has an influence on its adoption by older adults [20]. The perception and experience of stigma with the use of smart homes may result in older adults’ rejection of potentially useful tools to support their goals.

Discrimination is “the practice of unfairly treating a person or a group differently from other people or groups of people” [46] and is often the result of unfair and negative beliefs. Ageism (negative attitudes toward aging or older people), ableism (negative attitudes toward people with disabilities and their potential achievements), and mentalism (negative attitudes toward people with mental health or cognitive disorders) are all causes of discrimination. Discrimination may negatively influence what and how smart home technologies are developed. The choice of technology functions to create or the goals achievable by the technology may be informed by unfair beliefs about older people or people with disabilities. The lack of available data sets representing diverse age groups and abilities to inform AI development for these applications may be reflective of negative attitudes and contribute to the considerable problem of AI bias. What is considered normal or healthy may be determined by a small subset of people and their biases (eg, industry) [41]. Consequently, the developed functions or applications may not work for older adults with disabilities, or the decisions made by AI algorithms may not reflect the decisions of these users [41]. The inclusion of AI may not make decision-making more objective, and thus, predictions or recommendations should be used as guidance rather than as definitive decisions. Furthermore, even if technology is available, it may not be offered to older adults. This may be because caregivers, health care providers, or others may believe that older adults are not interested in it, are incapable of learning to use it, or will not benefit as much from it as younger people.

### Equity of Access

Equity of access to beneficial support that may enable health and well-being is a concern of justice. Being equitable may involve “treating people or distributing resources differently, when people are in different situations and unequal treatment or distribution creates an equal outcome” [27]. A concern is whether the availability of smart home technology with its associated costs will only benefit people who can afford to pay for it [19,25,33]. Inequitable access may result in older adults who are unable to pay (or without family support to pay) being excluded from the benefits of smart homes, experiencing poorer health and well-being, and being further socially excluded. At a societal level, this may deepen the existing digital divide, whereby only privileged groups benefit from the use of digital tools and broad adoption is restricted. Nonetheless, the cost of smart home technology is anticipated to be driven down as it becomes more available and prevalent (though initially for those who can pay) and technology production costs decrease. In recent years, technology to support health and social care for the growing older adult population has become an important development topic for industry.

Government policy makers have also considered the topic critical in policy actions to ensure the health and well-being of citizens and the sustainability of funding to support care systems. In managing resources and spending priorities, there is a need for consideration and evaluation of the cost-effectiveness of smart home implementation for older adults with frailty and the intended goals [19]. Policies that allow for faster uptake of smart home technology by everyone may require more insurance-based reimbursements in health care plans [25]. Notably, smart home technology was determined to be more acceptable to older adults if they were paid for by family or the government [18].

## Proactive and Ongoing Ethics Analysis and Management

### Overview

Consideration of ethical issues and ethical practices is increasingly viewed as core in development, evaluation, and implementation activities for assistive and rehabilitation technology, which includes smart homes [47]. Ethics is relevant to the (1) development (including conception) and implementation of smart homes to align outputs and outcomes with our ethical, social, and cultural values and (2) processes for development, evaluation, and implementation of smart homes. Existing processes entailing ethical considerations that are relevant to smart homes include approvals for ethical research conduct and regulatory approvals addressing safety, effectiveness, and standards compliance for product and service transfer to market. These processes are highly focused and may not thoroughly reflect the broader questions of what we develop and implement; whether these innovations align with our values; and the ethical implications of innovations (as in 1) and how we develop, evaluate, and implement smart homes and whether our processes are consistent with these values (as in 2).

The success of smart home technology will ultimately be adoption, sustained use that facilitates health and well-being outcomes of older adults and caregivers, and increased sustainability of health and social care systems. The concepts within the 6 domains presented have been shown in research to influence adoption and use of smart homes. Smart homes may have a greater likelihood of success with committed proactive and comprehensive analysis, anticipation, and management of ethical concerns. For users with frailty, these processes need especially to reflect and accommodate their unique circumstances throughout the course of projects. Older adults with frailty may require more nuanced consideration with regard to technology use owing to their heightened vulnerability resulting from limitations in physical and cognitive abilities, experiences of mental health concerns and isolation, and reliance on others. Frailty is a complex condition necessitating management through an interdisciplinary clinical approach alongside older adults and their caregivers. This scenario leads to greater complexity in ethical analysis. Development and implementation may be enhanced through ethically aligned practices that place users and their contexts and usability goals at the center of activities such that outputs are usable and accessible by users in various living situations and market and funding systems. We emphasize ongoing analysis and management as the conditions and information related to previous decisions may change over time. A mindset that embraces potential unknowns and promotes reevaluation and course correction if circumstances change may enable better outcomes.

A lack of consideration and mitigation of ethical issues may result in several negative outcomes. Unfavorable perceptions from the public regarding technology and its potential uses and benefits may lead to the rejection of technology-based solutions or the removal of public resources for future research and development. This may result in the loss of opportunities for future implementation to benefit users. Errors or differences in understanding or expectations with AI and other technologies may result in inappropriate policies and legislation and again halt potentially important progress [41]. The adoption of appropriate measures for oversight of technology using AI requires ethical considerations and should be established before implementation and use [44]. There may be amplification of stigma and discrimination related to older people, disability, or assistive technology use, which may result in harm or unrealized opportunities. Negative perceptions of technology from poor consideration of ethical issues may result in a lack of investment from business developers who may be needed to commercialize technology or lack of funding from public funders or private insurers to support access for use [48].

### Recommendations

To facilitate proactive and ongoing analysis and management of ethical concerns, we recommend collaboration among all stakeholders. This includes older adults; members of their support ecosystems; and the research, technical development, clinical, and industry communities in the field of smart home technology. This collaboration may be achieved through their inclusion in workshops and project teams. It is essential to cocreate a framework and associated resources and tools to support its implementation. The framework may include 4 elements to be discussed and elaborated on (Textbox 1).

**Elements of a framework to facilitate the proactive and ongoing analysis and management of ethical concerns.**

- A set of conceptual domains, such as those discussed in this paper, along with a tool consisting of reflective questions to guide the ethical deliberation of these domains

Systematic and standardized consideration of these domains across stages of a project from conception, development, evaluation, and implementation to sustained use is recommended as part of a comprehensive strategy. Reflective questions, potentially applying a Socratic approach for analyzing ethical implications [19,22], may be compiled as a tool. The tool may be used to examine ethical values for the technology and functions offered, what the technology may be used for or how it may be used, what is required for use, and the potential expected and unexpected outcomes. The analysis may cover personal, interpersonal, group, institutional, and societal levels of implications [41].

- Resources comprising strategies and guidance for the planning and reporting of ethical analysis throughout project phases

These resources may outline detailed strategies and guidance to be used at the start of and throughout projects to reflect on, anticipate, identify, define, deliberate, and mitigate real and potential ethical issues before and if they arise. New methods, guidelines, and checklists to support planning and reporting to enable transparency of ethical analyses during development, evaluation, and implementation processes, especially as they relate to decisions made throughout project phases, may need to be developed.

- Training resources to support leadership, literacy, and competency in teams for the analysis and management of ethical concerns

Leadership, collective team responsibility, and a culture that values the analysis and management of ethical concerns need to be promoted. Cross-disciplinary knowledge and skill development for researchers and practitioners in the clinical and technical sciences, industry members, and others may be essential to support literacy and competency in ethical analysis and management as part of smart home development, evaluation, and implementation. Contributing to the collaborative process, team members also need to develop knowledge and skills to meaningfully engage with older adults, their support ecosystems, and the public to fully include them in teams.

- Training resources for older adults with frailty, their support ecosystems, and the public to support their awareness and participation in teams and ethical analysis processes

These stakeholders are essential team members and prospective users of smart homes, and therefore, commitment and strategies to ensure their full inclusion are critical. Raising awareness of smart homes and their potential benefits and harms and enhancing knowledge and skills regarding ethics and ethical analysis are important to support critical and realistic assessment of technology for adoption, use, and provision of feedback to developers or providers. For older adults and their support ecosystems, knowledge and skill development may focus on strategies to communicate and advocate for their needs. Resources on technology and ethical analysis need to be easy to understand to promote knowledge exchange and learning.

## Conclusions

Successful adoption and sustained use of smart homes in the management of frailty in older adults have thus far been limited. Older adults with frailty require nuanced consideration when incorporating technology into their care because of their complex health and social status and vulnerability. Unmitigated ethical concerns are important factors restricting older adults and their support ecosystems from benefiting from the use of smart home technology. Applying a proactive and ongoing ethics analysis and management approach from development, evaluation, and implementation to sustained use is important for success. We recommend the development of a framework along with educational resources and analysis tools, cocreated by older adults, members of their support ecosystems, and other stakeholders, to support the implementation of this approach. Within this framework, consideration of a range of conceptual domains derived from bioethics and technology ethics is key: (1) privacy and security, (2) individual and relational autonomy, (3) informed consent and supported decision-making, (4) social inclusion and isolation, (5) stigma and discrimination, and (6) equity of access. Smart homes may have a greater likelihood of accommodating users and their contexts with committed and comprehensive analysis, anticipation, and management of ethical concerns that reflect the unique circumstances of these users. Smart home technology use may then achieve its desired individual, societal, and economic outcomes and serve as a solution to support health; well-being; and responsible, high-quality care.

## Abbreviations

AI: artificial intelligence
